# Heme-Peroxidase 2, a Peroxinectin-Like Gene, Regulates Bacterial Homeostasis in *Anopheles stephensi* Midgut

**DOI:** 10.3389/fphys.2020.572340

**Published:** 2020-09-08

**Authors:** Parik Kakani, Lalita Gupta, Sanjeev Kumar

**Affiliations:** ^1^Molecular Parasitology and Vector Biology Laboratory, Department of Biological Sciences, Birla Institute of Technology and Science (BITS), Pilani, India; ^2^Department of Biological Sciences, Indian Institute of Science Education and Research (IISER), Bhopal, India; ^3^Department of Zoology, Chaudhary Bansi Lal University, Bhiwani, India; ^4^Department of Biotechnology, Chaudhary Bansi Lal University, Bhiwani, India

**Keywords:** *Anopheles stephensi*, midgut, bacteria, heme-peroxidase, homeostasis, vector competence

## Abstract

The dynamic nature of mosquito gut microbiome is associated with different stages of development and feeding behaviors. Therefore, mosquito gut harbors a wide range of endogenous microbes that promote numerous life processes such as, nutrition, reproduction and immunity. In addition, gut microbiota also play an important role in the regulation of *Plasmodium* (malaria parasite) development. Thus, understanding the mechanism of microbial homeostasis in mosquito gut might be one of the strategies to manipulate malaria parasite development. In the present study, we characterized a 692 amino acids long secreted midgut heme-peroxidase 2 (AsHPX2) in *Anopheles stephensi*, the major Indian malaria vector. The presence of putative integrin binding motifs, LDV (Leu–Asp–Val), indicated its peroxinectin-like nature. Our phylogenetic analysis revealed that AsHPX2 is a Culicinae lineage-specific gene. RNA interference (RNAi)-mediated silencing of AsHPX2 gene significantly enhanced the growth of midgut bacteria in sugar-fed mosquitoes against sham-treated controls. Interestingly, blood-feeding drastically reduced AsHPX2 gene expression and enhanced the growth of midgut bacteria. These results revealed a negative correlation between the expression of AsHPX2 gene and gut bacterial growth. We proposed that AsHPX2, being a mosquito-specific gene, might serve as a “potent target” to manipulate midgut microbiota and vector competence.

## Introduction

Newly emerged mosquitoes are born with a limited energy reserve and therefore, they start feeding the nectar to replenish energy in order to power the flight for swarming, mating and blood-seeking activities. The initial feeding behavior contributes to the establishment of the microbial community in the mosquito midgut. The midgut microbes facilitate food digestion, metabolism, detoxification and the development of immunity (Gusmão et al., 2007; Minard et al., 2013; Kajla et al., 2015).

Interestingly, the dynamic nature of gut microbiota is also associated with different stages of mosquito development as well as their nutritional conditions. For example, in sugar-fed mosquito midguts cellulose degrading bacteria predominate, however, the bacteria facilitating blood digestion are more common in blood-fed midguts (de Gaio et al., 2011; Wang et al., 2011; Minard et al., 2013). In addition, the microbial interaction with mosquito midgut immunity also plays a pivotal role in the biological outcome of this symbiotic association. Thus, microbial homeostasis is managed by numerous immune mechanisms such as the production of lysozyme, reactive oxygen species (ROS), reactive nitrogen species (RNS), and antimicrobial peptides (AMPs) (Luckhart et al., 1998; Graca-Souza et al., 2006; Peterson and Luckhart, 2006; Brennan et al., 2008; Nishikori et al., 2009; Login et al., 2011; Wang et al., 2011).

It is also noteworthy to mention that the midgut microbiota also regulates *Plasmodium* development (Dong et al., 2009; Cirimotich et al., 2011). It was evident from numerous studies where co-feedings of either live or heat-killed bacteria decreased parasites number (Dong et al., 2009). Moreover, the parasite load also increased in antibiotics fed mosquitoes. Interestingly, the bacteria-induced ROS in mosquito midgut suppressed *Plasmodium* development (Beier et al., 1994; Dong et al., 2009; Cirimotich et al., 2011). Thus, we believe that the manipulation of midgut bacteria or mechanisms regulating bacterial homeostasis might be promising to alter the vectorial capacity (the property to support parasite development) of mosquito.

Heme-peroxidases regulate antibacterial pathways in mammals and insects. They catalyze H_2_O_2_-dependent oxidation of halides that, in turn, inhibit microbial metabolism and growth (Ha et al., 2005a; Klebanoff, 2005; Kumar et al., 2010; Kajla et al., 2016a). In addition, heme-peroxidases also catalyze the crosslinking of mucins layer over the midgut epithelium to block the interaction of lumen bacteria with mosquito immunity. This cross-linked barrier-based mechanism creates a low immunity zone in midgut to support the growth of microbial community (Kumar et al., 2010; Kajla et al., 2016a). Thus, mosquito peroxidases play a dramatic role in bacteria homeostasis and immunity.

Our analyses of *Anopheles stephensi* genome revealed the presence of 18 heme-peroxidases that participate in important biological functions (Kajla et al., 2016b; Choudhury et al., 2019; Kakani et al., 2019). In this study, we characterized *An. stephensi* midgut heme-peroxidase 2 (AsHPX2) and explored its role in the regulation of bacterial homeostasis. Our findings will open new opportunities to exploit the peroxidase-mediated immunemodulatory mechanism in mosquito for manipulating malaria parasite development.

## Materials and Methods

### Ethics Statement

The use of experimental animals in this study was approved by the BITS, Pilani Institutional Animal Ethical Committee (Approval number IAEC/RES/18/02). Animal maintenance and experiments were performed in accordance with guidelines of the Committee for the Purpose of Control and Supervision of Experiments on Animals (CPCSEA), Government of India.

### Rearing of Mosquitoes

*Anopheles stephensi* mosquitoes were reared in an insectary at Birla Institute of Technology and Science, Pilani, Rajasthan. The insectary was maintained at 28°C, 80% relative humidity (RH) and 12 h light:dark cycle as described before (Dhawan et al., 2017; Kajla et al., 2017). Adult mosquitoes were regularly fed on 10% sucrose solution. For colony propagation, 4- to 5-day-old females were fed on anesthetized mice and their eggs were collected in moist conditions. The hatched larvae were floated in water to continue the life cycle and fed on a 1:1 mixture of dog food (Pet Lover’s crunch milk biscuit, India) and fish food (Gold Tokyo, India) (Gupta et al., 2017; Choudhury et al., 2019; Kakani et al., 2019).

### Retrieval of Heme Peroxidase AsHPX2 Gene From *An. stephensi* Genome

*Anopheles gambiae* AgHPX2 gene (AGAP009033) was blast searched against the partially annotated genome of *An. stephensi* (taxid: 30069) to retrieve its ortholog. The best matching *An. stephensi* contig 7145 (SuperContig KB664566 and Ensembl identifier ASTE003848) was analyzed using Augustus software to identify the full-length putative AsHPX2 gene as before (Stanke et al., 2008; Choudhury et al., 2019; Kakani et al., 2019). AsHPX2 and AgHPX2 gene sequences were aligned to design gene-specific primers. The list of these primers is provided in Supplementary Table S1.

### Cloning, Sequencing and Phylogenetic Analysis of AsHPX2

Full-length AsHPX2 gene was PCR amplified from *An. stephensi* midgut cDNA template using Phusion High-Fidelity DNA Polymerase (Thermo scientific, #F-530S) and F4R5 primers. PCR was initiated at 98°C for 30 s followed by 35 cycles at 98°C for 10 s, 62°C for 30 s and 72°C for 2 min. The final extension was carried at 72°C for 10 min. The amplified product was purified and sequenced at Eurofins genomics. The sequence identity of full-length AsHPX2 cDNA was confirmed through general BLAST and submitted to NCBI Genbank (accession numbers: KY363390).

The 5′ upstream region of AsHPX2 gene was analyzed by Augustus and JASPAR/MatInspector software to mark the putative promoter and regulatory sequences, respectively as before (Cartharius et al., 2005; Stanke et al., 2008; Mathelier et al., 2014; Kajla et al., 2016b; Kakani et al., 2019). The search criteria for these analyses were restricted to the transcription factors from insect family with minimum 80% similarity.

To analyze the evolutionary relationship of AsHPX2 protein, phylogenetic tree was constructed using the neighbor-joining (NJ) method implemented in MEGA 5.2 program as before (Saitou and Nei, 1987). The sequences of different animal heme peroxidases, implemented to construct the tree, were downloaded from NCBI and listed in Supplementary Table S2. The branching pattern reliability of the phylogenetic tree was tested by 1,000 bootstrap replicates.

### In Silico Analysis of AsHPX2 Protein

Conserved domains in AsHPX2 protein were identified with the help of Conserved Domain Database (CDD) as well as SMART database (Letunic et al., 2014; Marchler-Bauer et al., 2015). The putative signal peptide in AsHPX2 protein was analyzed using SignalP and Phobius software (Käll et al., 2007; Petersen et al., 2011). The putative active sites and 3D structure of AsHPX2 protein was analyzed using Phyre^2^ and TASSER software (Kelley et al., 2015; Yang et al., 2015). The quality of the predicted AsHPX2 model was assessed by TM-score (Zhang, 2008).

### Collection of Different Mosquito Development Stages and Tissues

Different developmental stages of *An. stephensi* such as, eggs, first to fourth instar larvae, pupae, adult males or females were pooled and collected separately in RNAlater (Qiagen) and stored at −80°C. The midguts or carcasses (rest of the body parts after removing midguts) from sugar- or blood-fed females were also collected in a similar way. In some experiments, mosquitoes, as well as dissected midguts, were surface sterilized by rinsing them thrice, each for 5 s, in absolute ethanol and stored in RNAlater at −80°C (Gupta et al., 2009; Kumar et al., 2010; Wang et al., 2011).

### RNA Isolation, cDNA Preparation and Analysis of Gene Expression

Total RNA was isolated from mosquito tissues by RNAeasy mini kit (Qiagen) and the first-strand cDNA was synthesized using Quantitect reverse transcription kit (Qiagen) that included a genomic wipeout treatment step to remove genomic DNA contamination. Since, the central gene of interest in this study is intronless, therefore, to insure no gDNA contamination in RNA preparations we included a negative RT control (i.e., a cDNA preparation using DNase-treated RNA and all reaction components except the RT enzyme) in the qPCR analyses. The IQ5 multicolor real-time PCR detection system (Bio-Rad) was used to analyze the relative mRNA expressions of AsHPX2 gene and the bacterial load was determined by the amplification of 16S rRNA gene. The sequences of all the mosquito gene primers and universal 16S primers are listed in Supplementary Table S1. The ribosomal protein subunit S7 mRNA was used as an internal loading control as before (Kumar et al., 2010; Dixit et al., 2011; Kakani et al., 2019). PCR cycle parameters were following: 95°C for 5 min, 40 cycles each at 94°C for 20 s, 55°C for 30 s, and 72°C for 50 s. Fluorescence was read at 72°C after each cycle. The final extension was carried at 72°C for 10 min and then subjected to a melting curve to confirm the identity of PCR product. The end product was run on a gel to confirm the specificity of primers. Relative mRNA levels of various genes were calculated using ^ΔΔ^Ct method as before (Livak and Schmittgen, 2001; Kumar et al., 2010; Kakani et al., 2019). The PCR amplification efficiency was determined with LinRegPCR as described elsewhere (Ramakers et al., 2003).

### dsRNA Synthesis and Gene Silencing

A 450-bp fragment of AsHPX2 cDNA was amplified using F1R1 primers (Supplementary Table S1) and cloned into the pCRII-TOPO vector. Similarly, a 218-bp fragment of LacZ gene was amplified and cloned using the primers (5′ to 3′) F-GAGTCAGTGAGCGAGGAAGC and R-TATCCGCTCACAATTCCACA (Gupta et al., 2009; Kajla et al., 2017). These clones already have a T7 promoter site at one end thus, another T7 promoter was incorporated at the other end of the fragment by amplifying the insert with the primers: M13F-GTAAAACGACGGCCAGT and T7-M13R-CTCGAGTAATACGACTCACTATAGGGCAGGAAACAGCTA TGAC. PCR cycle parameters were following: 94°C for 5 min followed by 40 cycles each with 94°C for 30 s, 55°C for 30 s, and 72°C for 30 s. The final extension was carried at 72°C for 10 min. Amplicons were extracted from the gel with QIAquick Gel Extraction Kit (Qiagen) and used to synthesize dsRNA as per the instructions of MEGAscript RNAi kit (Ambion). dsRNA was purified with the help of Microcon YM-100 filter (Millipore) and finally concentrated to 3 μg/μl in DNase- and RNase-free water (Dhawan et al., 2017; Kajla et al., 2017; Kakani et al., 2019).

For gene silencing, 2-day-old female mosquitoes were injected with 69 nl of 3 μg/μl dsAsHPX2 (silenced) or dsLacZ (control) RNA into their thorax using a nanojector (Drummond). Four days after the injection, mosquitos were surface sterilized and their midguts were dissected as before (Gupta et al., 2009; Kumar et al., 2010; Wang et al., 2011).

### Statistical Analysis of the Data

All the data were expressed as a mean ± standard deviation. GraphPad Prism 5.0 software was used to analyze statistical significance between test and respective controls by performing Student’s *t*-test or one-way ANOVA (Motulsky, 1999). The data were considered significant if *p* < 0.05. Each experiment was performed at least thrice to validate the findings.

## Results

### Cloning and Characterization of AsHPX2 Gene

Putative AsHPX2 gene was identified in contig 7,145 of un-annotated *An. stephensi* genome as described in the Materials and Methods. Analysis of this contig by Augustus software predicted 2,868 bp full-length AsHPX2 gene containing 2,079 bp open reading frame (ORF), which encodes for a 692 amino acids long protein. The 5′-untranslated region (5′-UTR) and 3′-UTR of AsHPX2 gene were found to be 438 and 351 bp, respectively. The polyadenylation signal AATAAA was identified 331 bp downstream from the stop codon (Supplementary Figure S1).

Furthermore, the 5′ upstream region of AsHPX2 gene was analyzed by JASPAR and MatInspector software to identify putative transcription factor binding motifs (TFBM) in its promoter region. Our analyses indicated the binding sites for some major transcription factors such as, GATA (pnr), Rel1, AP-1 (a Jun/Fos dimer) and Broad complex (Br-C) (Supplementary Figure S1) that indicated a tissue-specific immune role of AsHPX2 in a way similar to other insect genes (Senger et al., 2006; Zhu et al., 2007; Garver et al., 2013; Kajla et al., 2016b; Zakovic and Levashina, 2017; Kakani et al., 2019).

**FIGURE 1:**
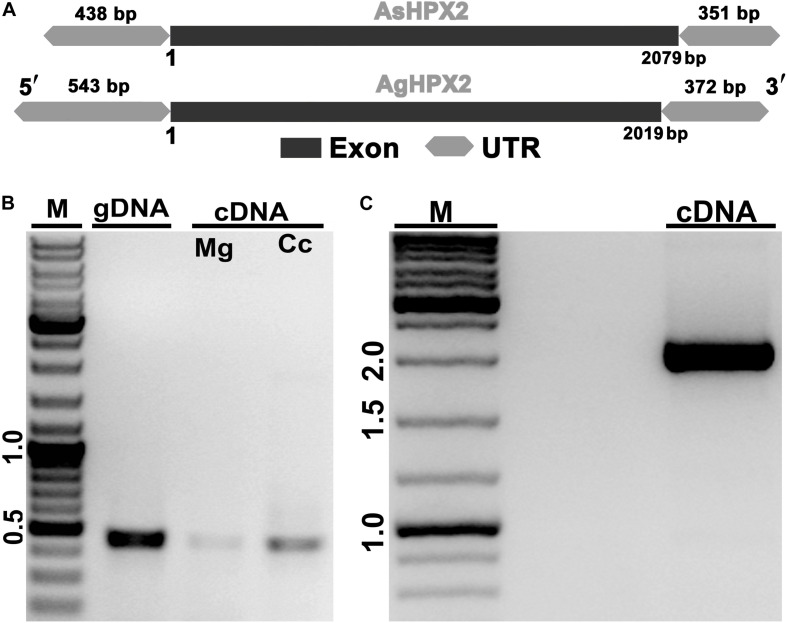
Molecular identification of AsHPX2 gene. **(A)** Genomic organization of AsHPX2 gene: both AgHPX2 (2,019 bp) and AsHPX2 (2,079 bp) genes have single exon. The 5′ and 3′ UTRs of AsHPX2 gene are 438 and 351 bp, respectively. However, 5′ and 3′ UTRs of AgHPX2 gene are 543 and 372 bp, respectively. **(B)** PCR amplification of 450 bp partial segment of AsHPX2 gene using F1R1 primers from genomic DNA (gDNA), midgut (Mg) or carcass (Cc) cDNA template. **(C)** PCR amplification of full-length AsHPX2 gene from cDNA template using F4R5 primers. M represents the DNA ladder in kb.

The predicted AsHPX2 cDNA sequence was aligned with *An. gambiae* AgHPX2 (AGAP009033) gene to design gene-specific primers (Supplementary Table S1) and F4R5 primers set amplified ∼2 kb full-length gene from *An. stephensi* cDNA (Figure 1). The PCR product was sequenced and blast search revealed its 80% identity with AgHPX2 gene (*e* value 3e^–107^). This sequence was submitted to NCBI (GenBank accession number: KY363390) and its alignment with the predicted *An. stephensi* genomic DNA revealed that AsHPX2 is an intron-less gene (Figure 1) in a way similar to its ortholog AgHPX2 (Giraldo-Calderón et al., 2015).

### Sequence and Domain Analysis of AsHPX2 Protein

**FIGURE 2:**
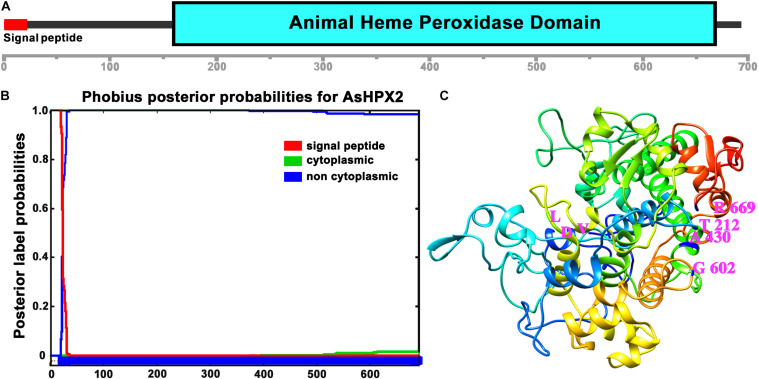
Domain organization of AsHPX2 protein. **(A)** The amino acid sequence of AsHPX2 protein was analyzed with the help of SMART program that revealed the presence of an animal heme peroxidase domain and *N*-terminal signal peptide. **(B)** The AsHPX2 protein sequence was analyzed using Phobius software that also revealed the presence of a signal peptide. **(C)** Three-dimensional structure of AsHPX2 protein was constructed by TASSER program that depicted the amino acids at active site (represented by letter symbols and numbers indicate their position). The integrin binding motif, LDV is oriented toward the surface of AsHPX2 protein.

Analyses of 76.9 kDa AsHPX2 protein by SMART program revealed that it is an animal heme-peroxidase (Figure 2). The presence of a 21 amino acids long signal peptide and signal peptidase cleavage site between Gly_21_ and Arg_22_ residues at *N*-terminal suggested that AsHPX2 is a secreted protein (Figure 2B). Phyre^2^ software analyses revealed the presence of 47% alpha helices in the secondary structure of AsHPX2 protein (Supplementary Figure S2) indicating that it is a globular protein as suggested by others (Pace and Scholtz, 1998; Kajla et al., 2016b). In addition, this protein also contains 10 heme-binding sites, 13 substrate-binding sites, three calcium-binding sites, and three homodimer interface sites (Supplementary Figure S2) in a way similar to other insect heme-peroxidases (Soudi et al., 2012; Kajla et al., 2016b; Bailey et al., 2017; Choudhury et al., 2019; Kakani et al., 2019). Three-dimensional structure of AsHPX2 protein (TM-score 0.70 ± 0.12) revealed the presence of catalytic quartet (Thr_212_, Ala_430_, Gly_602_, and Arg_669_), which is positioned toward the protein surface. Interestingly, the presence of a surface oriented triplet of Leu_530_, Asp_531_, and Val_532_ (LDV), which constitutes an integrin binding motif, indicated that AsHPX2 is a peroxinectin-like protein (Figures 2B,C) and might participate in cell adhesion as suggested by others (Johansson et al., 1995; Ruoslahti, 1996; Johanssen et al., 1999).

### Sequence Homology and Phylogenetic Analysis of AsHPX2 Protein

The sequence of AsHPX2 protein was aligned with heme-peroxidases from other organisms as listed in Supplementary Table S2 to analyze their sequence conservation. AsHPX2 shared 79 and 73% identity with *An. gambiae* AgHPX2 and *An. sinensis* AsnHPX2, respectively (Supplementary Table S2). On the other hand, it shared 57, 59, and 58% identity with *Aedes aegypti* AeHPX2, *Ae. albopictus* AaHPX2, and *Culex quinquefasciatus* CqHPX2, respectively. Interestingly, AsHPX2 protein shared only 34% identity with *Drosophila melanogaster* immune-related catalase (IRC), a heme-peroxidase that performs catalase cycle (Ha et al., 2005a,b). In addition, it shared less than 30% identity with human peroxidases (Supplementary Table S2). Evolutionary relationship of AsHPX2 protein with heme-peroxidases from other organisms (as mentioned in Supplementary Table S2) also revealed that mosquito HPX2s appeared in a single cluster and are diverged from *D. melanogaster* IRC (Supplementary Figure S3). Human peroxidases appeared in a separate cluster far away from mosquito HPX2 peroxidases.

### AsHPX2 Is Distinctively Expressed in Different Developmental Stages of Mosquito

**FIGURE 3:**
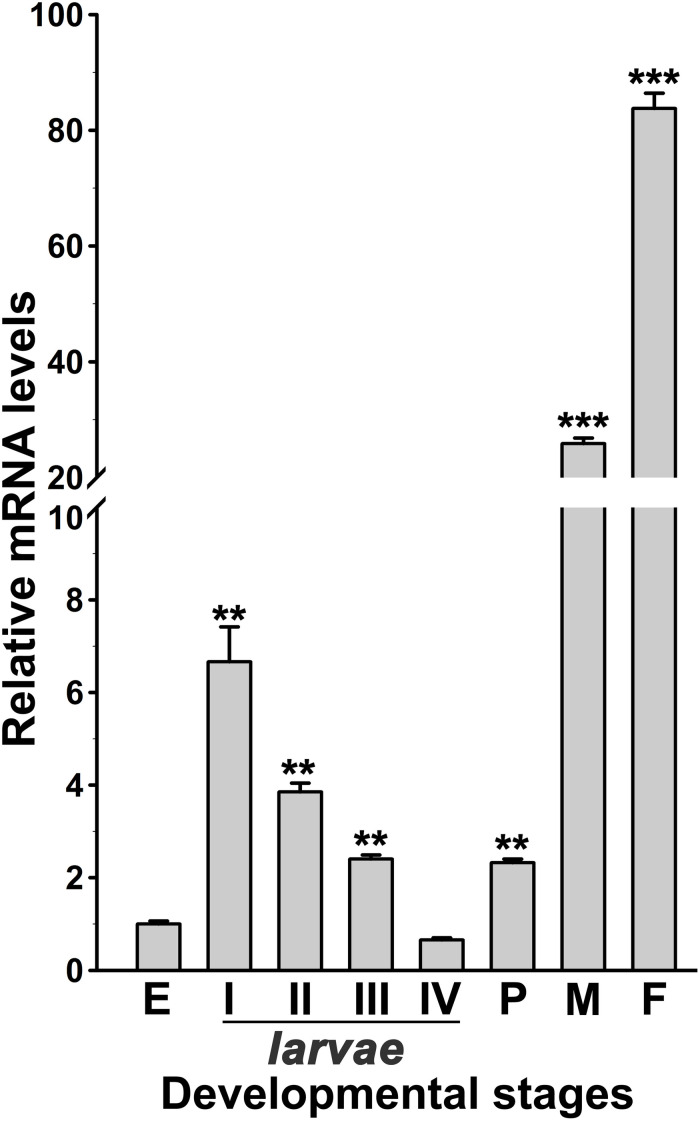
Analysis of AsHPX2 gene expression in different development stages of *An. stephensi*. Relative mRNA levels of AsHPX2 gene were analyzed in different stages of mosquito development against the mRNA levels of eggs, which were considered as 1.0. Significant differences (*p* < 0.001 or 0.01) are indicated by three or two asterisks, respectively. E, eggs; I–IV indicate different stages of instar larvae; *P*, pupae; *M*, males; *F*, females.

The expression of AsHPX2 gene in different developmental stages of *An. stephensi* was analyzed by qRT-PCR. AsHPX2 gene is expressed throughout all developmental stages of *An. stephensi* namely, eggs, first to fourth instar larvae, pupae, adult males or females (Figure 3). The relative mRNA levels of AsHPX2 gene were 6-fold, 4-fold, 2.5-fold, and 0.65-fold in first, second, third, and fourth instar larvae, respectively when compared to the eggs (*p* = 0.0087, *p* = 0.0024, *p* = 0.0030, and *p* = 0.0148, respectively). Furthermore, the relative AsHPX2 mRNA levels were 2-fold, 26-fold and 84-fold in pupae, adult males and females, respectively, against eggs (*p* = 0.0012, *p* = 0.0008, and *p* = 0.0005, respectively) (Figure 3).

### Blood-Feeding Down Regulates the Expression of AsHPX2 Gene

**FIGURE 4:**
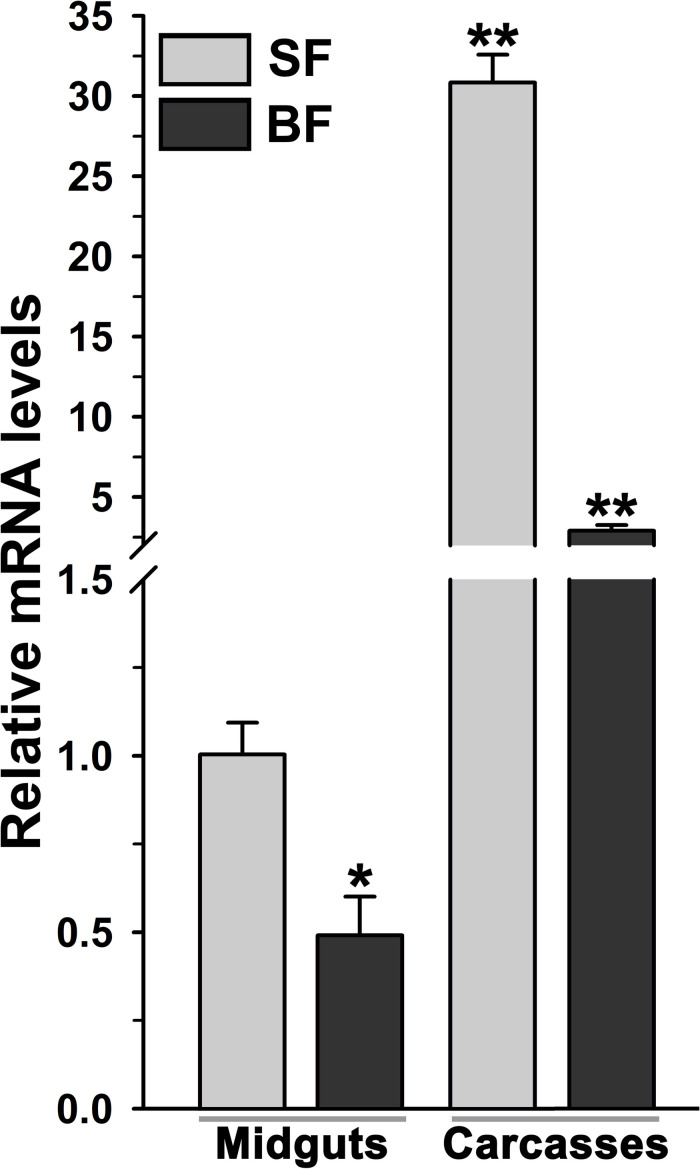
Analysis of mosquito body compartment-specific expression of AsHPX2 gene. Relative mRNA levels of AsHPX2 gene were analyzed in sugar-fed or 24 h post blood-fed mosquito midguts or carcasses. The relative levels of AsHPX2 were calculated against mRNA levels of sugar-fed midguts, which were considered as 1.0. Significant differences *p* < 0.01 or <0.05 are indicated by two or one asterisk, respectively.

**FIGURE 5:**
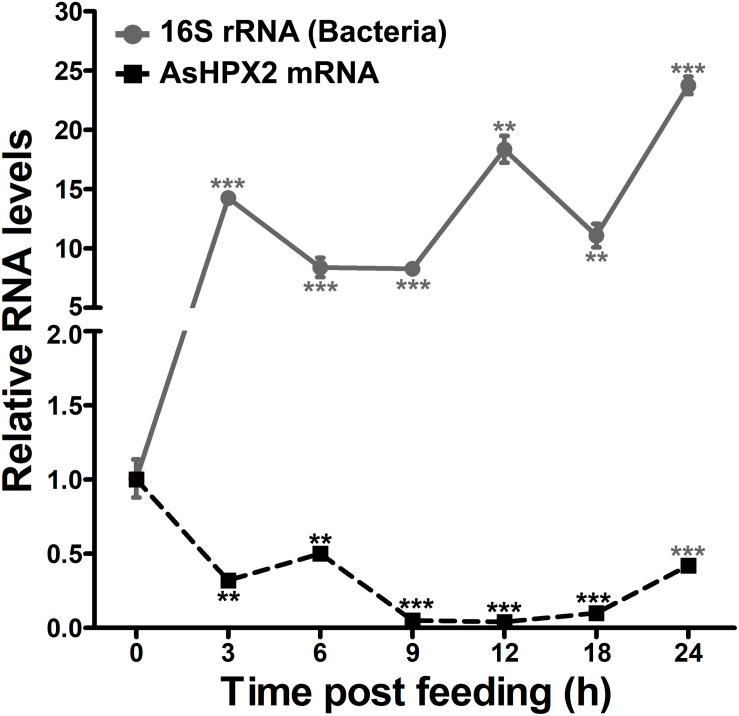
Expression kinetics of AsHPX2 gene in blood-fed midguts. Relative mRNA levels of AsHPX2 gene or 16S rRNA were analyzed in midguts collected at different time points after blood-feeding. The relative levels of AsHPX2 mRNA and 16S rRNA were calculated against the respective sugar-fed midguts, which were considered as 1.0. Significant differences (*p* < 0.001 or 0.01) are indicated by three or two asterisks, respectively.

The relative mRNA levels of AsHPX2 gene were analyzed in sugar- or 24 h post blood-fed midguts or carcasses to decipher its tissue-specific expression. The results presented in Figure 4 showed that AsHPX2 mRNA levels were 33-fold higher in sugar-fed carcasses than midguts (*p* = 0.0017). Moreover, its mRNA levels were reduced twofold (*p* = 0.0367) and 10-fold (*p* = 0.0020) in 24 h post blood-fed midguts and carcasses, respectively against their sugar-fed controls. In addition, the time kinetics also revealed that the midgut expression of AsHPX2 gene is down regulated after blood-feeding when compared to the sugar-fed controls (Figure 5).

The downregulation of AsHPX2 gene expression in blood-fed midguts might be explained by the presence of Z3 isoform of Br-C TFBM in its regulatory region (Supplementary Figure S1), which restricts tissue- and condition-specific expression of the gene (Von Kalm et al., 1994; Bayer et al., 1996; Chen et al., 2004; Zhu et al., 2007).

### AsHPX2 Regulates the Bacterial Homeostasis in Midgut

**FIGURE 6:**
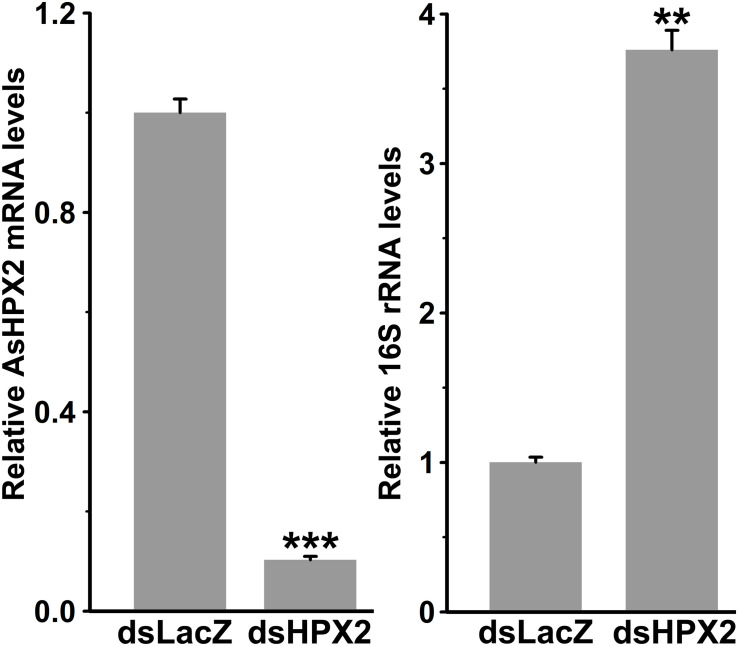
Relative levels of 16S rRNA in AsHPX2 silenced midguts. Mosquitoes were injected with dsLacZ (controls) or dsAsHPX2 (silenced) RNA and relative levels of AsHPX2 mRNA or 16S rRNA were analyzed in their midguts 4 days post-injection. Significant differences (*p* < 0.001 or 0.01) are shown by three or two asterisks, respectively.

The domain analyses revealed that AsHPX2 exhibits peroxinectin-like nature (has cell adhesive property and role in immunity) (Figure 2C and Supplementary Figure S2). Thus, we hypothesized that AsHPX2 gene might regulating bacterial homeostasis in mosquito midguts. To demonstrate this, AsHPX2 gene was silenced in the midguts of sugar-fed mosquitoes as described in Materials and Methods and its effect was evaluated on the levels of endogenous bacteria. Our analyses of AsHPX2 mRNA levels in controls and silenced midguts revealed 90% silencing of this gene (*p* = 0.0006, Figure 6). Furthermore, the analyses of 16S rRNA levels of endogenous bacteria in these samples indicated that their levels increased ∼4-fold in silenced midguts against controls (*p* = 0.0012, Figure 6). These results established the antibacterial role of AsHPX2 gene.

The silencing of AsHPX2 gene increased bacterial load in the midguts of sugar fed mosquitoes (Figure 6). Interestingly, our time kinetics study revealed that blood-feeding induced the growth of midgut bacteria (Figure 5) in a similar way as reported in our previous publications as well as by others (Kumar et al., 2010; Oliveira et al., 2011; Habtewold et al., 2016; Kajla et al., 2016a). However, on the other hand, there is an inverse correlation between the increased growth of bacteria and expression of AsHPX2 gene in blood-fed midguts (Figure 5). Thus, we believe that the down regulation of AsHPX2 gene in blood fed midguts (Figures 4, 5) augments the growth of midgut bacteria to support blood digestion.

**FIGURE 7:**
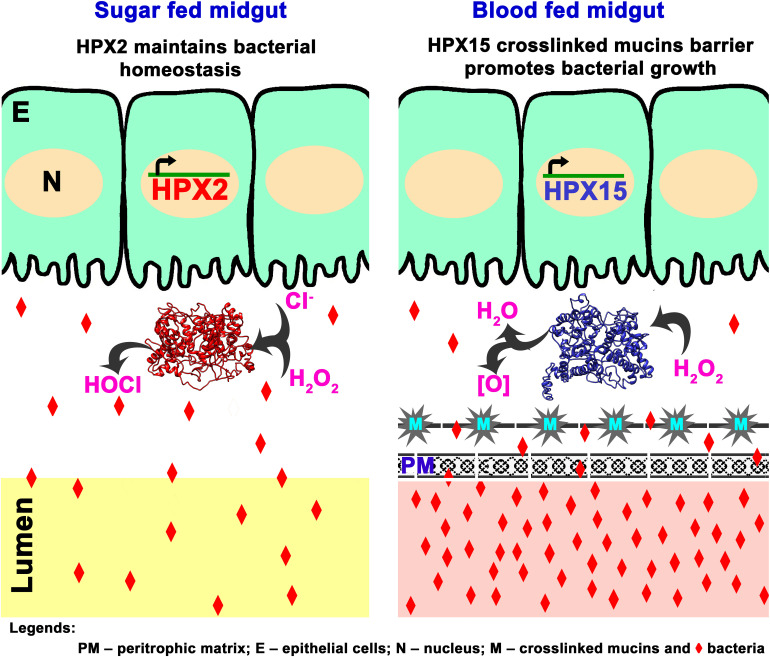
Proposed mechanism of AsHPX2 mediated bacterial homeostasis in *An. stephensi* midgut. In sugar-fed midguts, AsHPX2 catalyzes the halogenation cycle and produces hypochlorous acid (HOCl) to maintain the bacterial homeostasis. Blood-feeding suppresses the expression of AsHPX2 gene (Figure 5) and induces another heme-peroxidase AsHPX15 (Kumar et al., 2010; Kajla et al., 2016b), which catalyzes mucins crosslinking at the surface of midgut epithelium. Cross-linked mucins act like a barrier that blocks the recognition of bacteria by the immune-reactive epithelium. This mechanism creates a “low immunity zone” in this body compartment to promote the growth of bacteria that, in turn, facilitate blood digestion (Kumar et al., 2010; Kajla et al., 2015). Collectively, the reduced AsHPX2 expression and induced expression of HPX15 in blood-fed midguts create a fine-tuned balance between blood digestion and immunity.

Together, we proposed a putative model that describes the role of AsHPX2 gene in maintaining gut microbiome homeostasis as depicted in Figure 7. In brief, AsHPX2, a peroxinectin-like peroxidase limits the growth of bacteria in sugar-fed midguts most probable through the formation of hypochlorous acid (HOCl) produced from H_2_O_2_ and halides via halogenation cycle (Klebanoff, 1968; Klebanoff, 2005; Allen and Stephens, 2011). After a blood meal, AsHPX2 gene down-regulation (Figure 5) and up-regulation of another heme-peroxidase AsHPX15 that catalyzes the crosslinking of mucins barrier over the immune reactive midgut epithelium (Kumar et al., 2010; Kajla et al., 2015, 2016b) collectively promote the growth of endogenous bacteria to support various life processes such as digestion and reproduction (Oliveira et al., 2011; Kajla et al., 2015; Habtewold et al., 2016).

## Discussion

In this study, we characterized AsHPX2 gene from *An. stephensi* mosquito that encodes for a 692 amino acid long secreted protein. Interestingly, the intronless nature of AsHPX2 gene indicates that it has a tissue-specific expression with a high rate of evolution in a way similar to other intronless genes such as calmodulin-like gene NB1 and Poly(A) polymerase in human (Le et al., 2001; Shabalina et al., 2010). AsHPX2 has >70 and <60% amino acid identity with anopheline and Culicinae HPX2s, respectively. However, it shares <35% identity with human peroxidases and *D. melanogaster* IRC, a heme-peroxidase that performs catalase cycle (Ha et al., 2005a,b). In conclusion, these data indicated that AsHPX2 is a mosquito-specific gene. The presence of different TFBM in the regulatory region of AsHPX2 gene signifies its importance in mosquito biology. For example, GATA (pnr) and Br-C binding might explain its tissue-specific expression and down-regulation in blood-fed midguts, respectively in a way similar to other insect genes (Von Kalm et al., 1994; Bayer et al., 1996; Chen et al., 2004; Senger et al., 2006; Zhu et al., 2007; Kajla et al., 2016b; Kakani et al., 2019). It is noteworthy to mention that Br-C isoforms are activated in response to the blood-feeding induced ecdysone. Interestingly, out of four different isoforms of Br-C (Z1 to Z4), any one of them can circumstantially either induce or suppress a target gene (Von Kalm et al., 1994; Edgar, 2006; Zhu et al., 2007). For example, a robust expression of vitellogen gene is induced by Z2 isoform of Br-C in mosquito fat body within 24 h of blood-feeding. However, Z1 and Z4 isoforms of Br-C ensure the termination of vitellogen gene expression after 24 h post blood meal (Zhu et al., 2007). In summary, Br-C isoforms participate in the regulation of specific gene(s) in a coordinated and well-orchestrated way.

The presence of integrin binding motif (LDV) in AsHPX2 protein suggested its peroxinectin-like nature. Peroxinectins are invertebrate peroxidases that are involved in cell adhesion (Liu et al., 2004; Vizzini et al., 2013; Shanthi et al., 2014). Interestingly, the surface orientation of LDV motif in AsHPX2 protein seems to be facilitating its adhesion in a way similar to other adhesion molecules (Johanssen et al., 1999; Liu et al., 2005). Peroxinectins selectively bind to the bacteria and catalyze the formation of HOCl, an anti-bacterial molecule, through halogenation cycle (Klebanoff, 1968, 2005; Allen and Stephens, 2011). Interestingly, peroxinectins also bind superoxide dismutase (SOD), an enzyme that catalyzes the formation of H_2_O_2_ from superoxide anions, which, in turn, promotes the continuity of halogenation cycle (Holmblad and Soderhall, 1999; Johanssen et al., 1999; Choudhury et al., 2019).

Differential expression of AsHPX2 gene in different stages of mosquito development corroborates with the expression profile of some peroxidases (Mdes000915, Mdes001004, Mdes009520, and Mdes015930) across the developmental stages of Hessian fly *Mayetiola destructor* (Chen et al., 2016). Because peroxidases catalyze the crosslinking of biological matrices and regulate immune functions thus, their expression might reveal a direct association to the immune status and/or bacterial homeostasis during insect development. Direct evidence for this belief was reported in *An. gambiae* where a dynamic mosquito gut microbiome is associated with transitions in developmental stages and alteration of feeding behavior from the sugar diet to blood meal (Wang et al., 2011).

AsHPX2 gene silencing significantly enhanced the growth of midgut bacteria that revealed its antibacterial role in sugar fed mosquitoes. Therefore, the downregulation of AsHPX2 gene expression in blood-fed midguts is an important event and it is inversely correlated to the growth of bacteria. Because the bacteria play an important role in blood digestion thus, down-regulation of AsHPX2 gene creates a physiological condition in blood-fed midguts to facilitate digestion (Kumar et al., 2010; de Gaio et al., 2011; Kajla et al., 2015). Our results indicated that AsHPX2 is one of the important regulators that maintain homeostasis of endogenous bacteria in sugar- or blood-fed midguts. Interestingly, blood-feeding up-regulates the expression of another heme-peroxidase, AsHPX15 that catalyzes the crosslinking of mucins barrier over the immune reactive midgut epithelium to protect the growing bacteria (Kumar et al., 2010; Kajla et al., 2015, 2016b).

Thus, the systematic regulation of bacterial antagonistic (AsHPX2) and agonistic (AsHPX15) peroxidases create a low immunity zone in midgut for promoting the growth of bacteria, facilitating blood digestion and maintaining a fine balance between food digestion and immunity. Hence, the regulated expression of heme-peroxidases is one of the mechanisms that actively maintain bacterial homeostasis in mosquito midgut during distinctive nutritional conditions.

In conclusion, the identification of *Anopheles* genes that maintain microbial homeostasis in the midgut are of great value as they can be exploited to achieve dysbiosis of the mosquito gut ecosystem and hence, the vector competence. Overall, from the present study, it is clear that AsHPX2 is a mosquito-specific gene that plays an important role in midgut physiology and immunity. Hence, this gene can be a potent target to alter the bacterial community and regulating *Plasmodium* development.

## Data Availability Statement

The raw data supporting the conclusions of this article will be made available by the authors, without undue reservation.

## Ethics Statement

The animal study was reviewed and approved by BITS, Pilani Institutional Animal Ethical Committee.

## Author Contributions

PK, LG, and SK designed the experiments, performed expression kinetics of AsHPX2 and silencing experiments, analyzed the data, and wrote the manuscript. PK and SK collected samples to perform experiments. All authors approved the manuscript.

## Conflict of Interest

The authors declare that the research was conducted in the absence of any commercial or financial relationships that could be construed as a potential conflict of interest.
