# Supplementary material for: Heme-Peroxidase 2, a Peroxinectin-Like Gene, Regulates Bacterial Homeostasis in Anopheles stephensi Midgut
Source: Front Physiol. 2020 Sep 8;11:572340. doi: 10.3389/fphys.2020.572340 (PMC7506126; doi:10.3389/fphys.2020.572340)
Supplement: Supplementary file 1 [file Data_Sheet_1.pdf]

Supplementary Information

Heme-peroxidase 2, a peroxinectin-like gene, regulates bacterial homeostasis in *Anopheles stephensi* midgut

Parik Kakani[1,2], Lalita Gupta[1,3] and Sanjeev Kumar[1,4*]

[1]Molecular Parasitology and Vector Biology Laboratory, Department of Biological Sciences, Birla Institute of Technology and Science (BITS), Pilani, India.

[2] Present address: Department of Biological Sciences, Indian Institute of Science Education and Research (IISER), Bhopal, Madhya Pradesh, India.

[3]Department of Zoology, Ch. Bansi Lal University, Bhiwani, Haryana, India.

[4]Department of Biotechnology, Ch. Bansi Lal University, Bhiwani, Haryana, India.

*** Corresponding author**

Prof. Sanjeev Kumar

Department of Biotechnology, Chaudhary Bansi Lal University, Bhiwani, Haryana 127021 (India)

Email: sanjeevnih@gmail.com

This file contains:

3 Supplementary Figures

2 Supplementary Tables

1 Complementary Figure

Figure S1

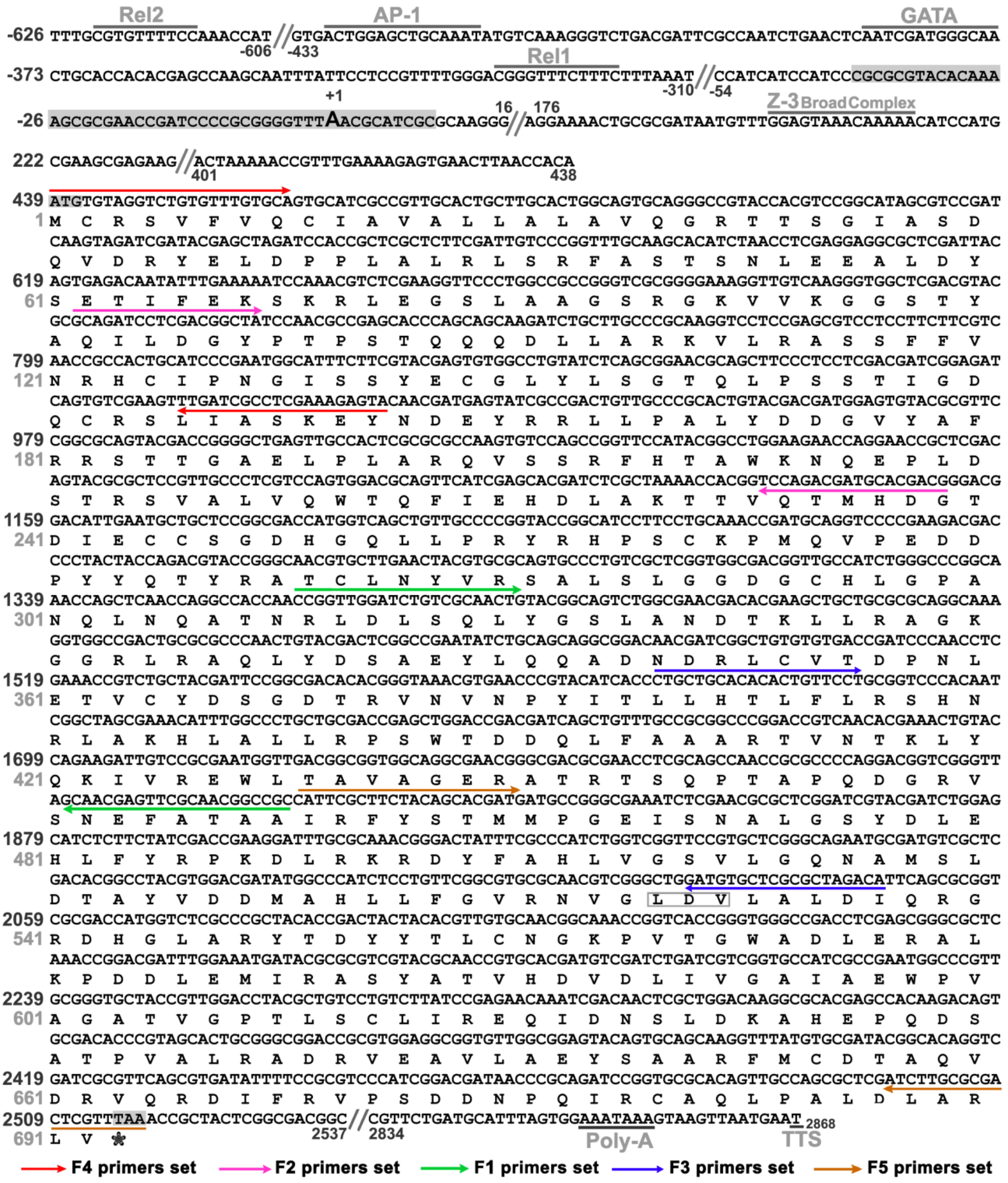

Supplementary Figure S1: The nucleotide and deduced amino acid sequence of AsHPX2 gene. The complete AsHPX2 cDNA (2868 bp) encodes for a 692 amino acids long protein (residue number mentioned on the left in gray). Primer sets used in this study as mentioned in Supplementary Table S1 are depicted in various color codes. Binding sites for various transcription factors such as GATA/Rel1, Broad complex and AP-1 are depicted in the regulatory region of AsHPX2 gene as revealed by JASPAR and MatInspector software. Transcription start site (TSS) is denoted by +1. The promoter, protein start codon ATG and stop codon TAA are highlighted in gray. The integrin binding motif LDV is boxed gray. Polyadenylation (Poly-A) and transcription termination site (TTS) are underlined.

Figure S2

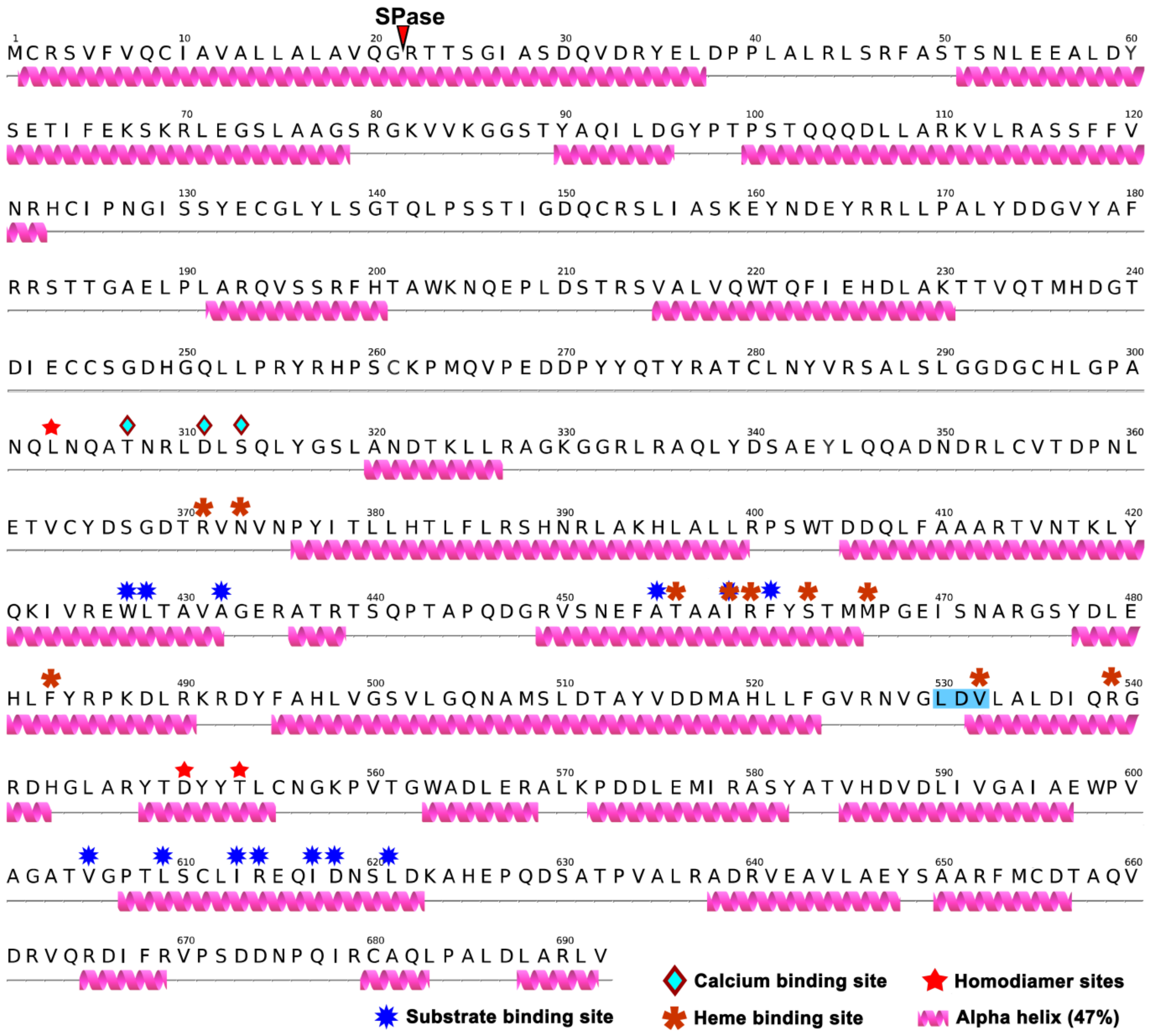

Supplementary Figure S2: Secondary structure of AsHPX2 protein. AsHPX2 protein contains several alpha helices (represented by spirals) as predicted by Phyre[2] software. The signal peptidase (SPase) cleavage site is indicated by an arrowhead. Various conserved binding sites in the protein are represented by characteristic symbols.

Figure S3

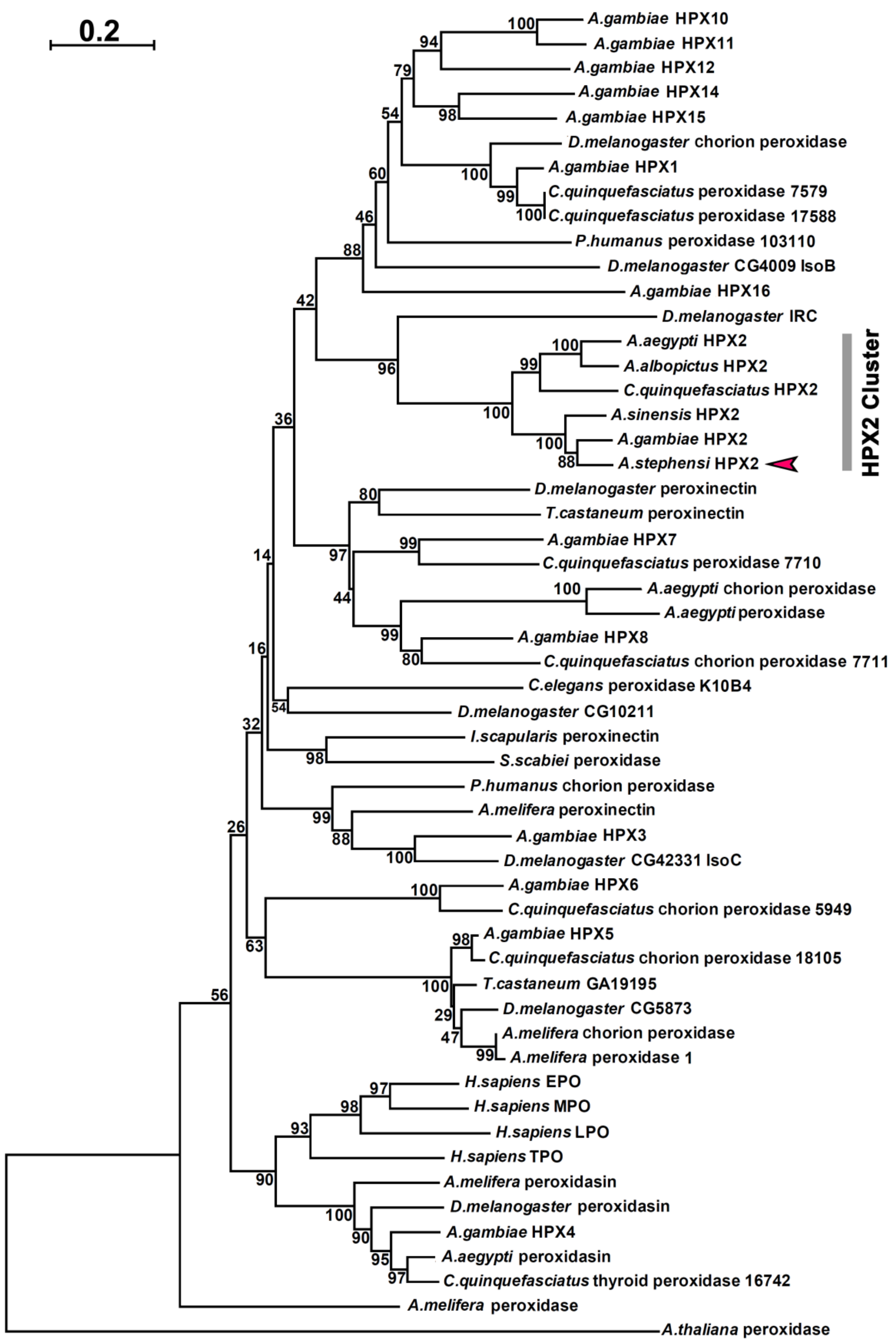

Supplementary Figure S3: Phylogenetic analysis of AsHPX2 protein. The Neighbour-joining (NJ) method was used to construct the phylogenetic tree of AsHPX2 with other heme peroxidases (HPXs) retrieved from different organisms as listed in Supplementary Table S2. Arrowhead indicates AsHPX2 protein that represents the part of the mosquito-specific cluster. The scale bar represents base substitutions per site. The numbers on branches represent the percent of 1000 bootstraps.

Supplementary Table S1: List of *An. stephensi* primers. Primer sets used to amplify different *An. stephensi* gene fragments are mentioned below.

Primer Sets	Sequence (5′-3′)	Amplified PCR product (bp)		Purpose	References
		cDNA template	gDNA template		
AsHPX2F1<br>AsHPX2R1	CCGGTTGGATCTGTCGCAAC<br>GCGGCCGTTGCGAACTCGTTGC	445	445	Cloning, dsRNA preparation	Present study
AsHPX2F2<br>AsHPX2R2	GCAGATCCTCGACGGCTA<br>CGTCGTGCATCGTCTGGA	443	443	qPCR	Present study
AsHPX2F3<br>AsHPX2R3	CTGCTGCACACACTGTTCCT<br>TGTCTAGCGCGAGCACATC	472	472	Sequencing	Present study
AsHPX2F4<br>AsHPX2R4	ATGTGTAGGTCTGTGTTTGTGCA<br>TACTCTTTCGAGGCGATCAA	482	482	Sequencing	Present study
AsHPX2F5<br>AsHPX2R5	TTCGCTTCTACAGCACGATG<br>TTAAACGAGTCGCGCAAGAT	706	706	Sequencing	Present study
16S rRNA F<br>16S rRNA R<br>(universal primers)	TCCTACGGGAGGCAGCAGT<br>GGACTACCAGGGTATCTAATCCTGTT	467	467	qPCR	(Kumar et al., 2010)
S7 F<br>S7 R	GGTGTTCGGTTCCAAGGTGA<br>GGTGGTCTGCTGGTTCTTATCC	487	600	PCR internal loading controls	(Dixit et al., 2011; Kakani et al., 2019)

Supplementary Table S2: List of peroxidases from diverse organisms selected for phylogenetic analysis. The heme peroxidases from various organisms were obtained from NCBI and Vectorbase databases.

**Organisms name**	**Peroxidase nomenclature**	**Gene ID/ Accession Number**	**Percentage identity with AsHPX2 protein**
An. stephensi	AsHPX2	KY363390	____
An. sinensis	AsiHPX2	ASIS008239	72.6
An. gambiae	HPX1	AGAP010734	29.4
	HPX2	AGAP009033	77.6
	HPX3	AGAP003714	25.8
	HPX4	AGAP007237	23.9
	HPX5	AGAP000051	22.5
	HPX6	AGAP003502	27.9
	HPX7	AGAP004036	25.2
	HPX8	AGAP004038	26.7
	HPX10	AGAP013282	27.8
	HPX11	AGAP010899	27.3
	HPX12	AGAP010735	26.0
	HPX14	AGAP010810	29.9
	HPX15	AGAP013327	30.0
	HPX16	AGAP011216	28.6
Aedes aegypti	Chorion Peroxidase	AAEL004386	20.7
	HPX2	AAEL013171	56.1
	Peroxinectin	AAEL003612	20.2

	Peroxidasin	AAEL000342	23.3
Aedes albopictus	HPX2	AALF004168	58.5
Culex quinquefasciatus	Chorion Peroxidase	CPIJ007711	24.2
	Chorion Peroxidase	CPIJ018105	22.7
	Chorion Peroxidase	CPIJ005949	27.2
	Peroxidase	CPIJ007579	29.1
	Peroxidase	CPIJ017588	28.6
	Peroxidase	CPIJ007710	24.2
	Thyroid Peroxidase	CPIJ016742	23.6
	HPX2	CPIJ001764	57.1
Drosophila melanogaster	Peroxidase	CG4009	27.0
	Peroxidase	CG5873	21.8
	Peroxidase	CG10211	25.5
	Peroxidase	CG42331	28.7
	Peroxidase (IRC)	CG8913	33.8
	Chorion peroxidase	CG3477	30.0
	Peroxidasin	CG12002	23.4
	Peroxinectin	CG7660	22.2
Pediculus humanus corporis	Chorion peroxidase	PHUM184790	24.1
	Peroxidase	PHUM103320	23.6

Tribolium castaneum	Peroxidase	GA19195	24.1
	Peroxinectin	CG7660	25.1
Caenorhabditis elegans	Peroxidase	CELE_K10B4.1	22.5
Arabidopsis thaliana	Peroxidase1	AAA32849	10.0
Homo sapiens	Eosinophil peroxidase (EPO)	8288	26.1
	Myeloperoxidase (MPO)	4353	24.0
	Thyroid peroxidase (TPO)	7173	25.2
	Lactoperoxidase (LPO)	4025	23.9
Ixodes scapularis	Peroxinectin	ISCW002680	28.8
Apis mellifera	Peroxidase	LOC724541	26.6
	Chorion Peroxidase	LOC412013	24.3
	Peroxidasin	LOC413025	23.6
	Peroxnectin	LOC551544	25.4
	Peroxidase	LOC408953	23.6
Sarcoptes scabiei	Peroxidase	KPM11066	30.8

References

Dixit, R., Rawat, M., Kumar, S., Pandey, K. C., Adak, T., Sharma, A. (2011). Salivary gland transcriptome analysis in response to sugar feeding in malaria vector *Anopheles stephensi*. J. Insect Physiol. 57, 1399-406.

Kakani, P., Kajla, M., Choudhury, T. P., Gupta, L., Kumar, S. (2019). *Anopheles stephensi* dual oxidase silencing activates the thioester-containing protein 1 pathway to suppress *Plasmodium* development. J. Innate Immun. 11, 496-505.

Kumar, S., Molina-Cruz, A., Gupta, L., Rodrigues, J., Barillas-Mury, C. (2010). A peroxidase/dual oxidase system modulates midgut epithelial immunity in *Anopheles gambiae*. Science 327, 1644–1648.

Complementary Information

(a)

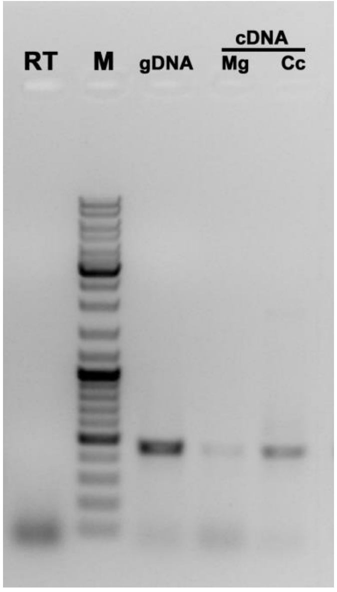

(b)

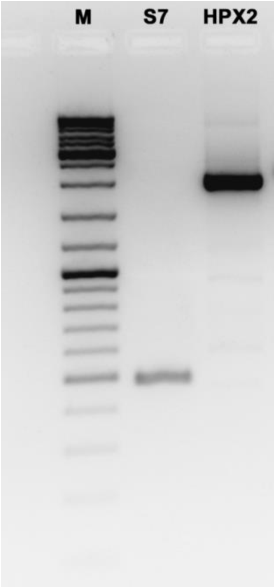

Complementary information 1: Original electrophoretic gel related to the PCR amplification of AsHPX2 gene (a) PCR product of AsHPX2 (from left: RT control (as mentoned in Materials and Methods); Marker (M); Genomic DNA (gDNA); Midgut (Mg-cDNA); Carcass (Cc-cDNA)) using F1R1 and (b) PCR product (from left: Marker (M); S7 (internal loading control); AsHPX2 (amplified with F4R5 primers) using cDNA as template. The internal control S7 is provided in the original gel is cropped in the final figure.
